# The Storm Before the Calm: Ablation of Premature Ventricular Complex Trigger for Incessant Ventricular Fibrillation

**DOI:** 10.19102/icrm.2021.120501

**Published:** 2021-05-15

**Authors:** Matthew J. Singleton, Prashant D. Bhave, Elijah H. Beaty, Natalie S. Bradford, S. Patrick Whalen

**Affiliations:** ^1^Section of Cardiology, Department of Internal Medicine, Wake Forest School of Medicine, Winston-Salem, NC, USA

**Keywords:** Catheter ablation, PVC, treatment-refractory, ventricular fibrillation

## Abstract

Ventricular tachycardia storm is associated with high mortality rates and is often refractory to treatment. Historically, few options for treatment have existed in cases when antiarrhythmic drugs fail. We report the case of a patient with incessant ventricular fibrillation (VF) in the postinfarction period that was triggered by premature ventricular contractions (PVCs) that persisted despite normal electrolytes, exclusion of ongoing ischemia, infusions of antiarrhythmic drugs, general anesthesia, full circulatory support with extracorporeal membranous oxygenation, and cardiac sympathetic denervation. Given that the VF appeared to be triggered consistently by a unifocal, short-coupled PVC (consistent with Purkinje fiber–mediated VF), we performed catheter ablation, after which point, the patient experienced no further PVCs or ventricular arrhythmia. This case serves as a reminder of three key teaching points. First, not all VF is created equal, with some cases being chiefly the result of a vulnerable substrate and others being best accounted for by frequent triggers. Second, examining the available electrocardiographic data and appropriately interpreting them can guide the selection of therapies up to and including catheter ablation for treatment-refractory VF. Third, full circulatory support greatly facilitates successful electroanatomic mapping and catheter ablation of unstable ventricular arrhythmias.

## Introduction

VAs account for an estimated 200,000 deaths in the United States per year.^1^ Despite their societal burden, an assessment of the risk of VAs remains imprecise,^2,3^ with the vast majority of events occurring in the low-risk population and the majority of high-risk patients not experiencing such events.^1^ Although implantable cardioverter-defibrillators are the mainstay of therapy for ventricular fibrillation (VF) and prevent sudden cardiac death, these devices are contraindicated (class IIIc) in the setting of incessant VAs.^4^ In patients with ongoing VAs despite antiarrhythmic drug therapy, catheter ablation has been described.^5,6^ However, because of the ephemeral, unpredictable nature of VF, data are not available to guide the approach to patient selection for catheter ablation. Here, we report a case of treatment-refractory VF storm wherein the patient was rescued by focal catheter ablation of the triggering PVC and discuss considerations in electrocardiographic (EGC) interpretation and patient selection.

## Case presentation

A 53-year-old man with no significant medical history presented with crushing chest pain. On his presenting ECG, he had an anterior ST-segment elevation. He was taken for emergent coronary angiography, which demonstrated that the culprit lesion was a 100% proximal left anterior descending artery occlusion. Percutaneous intervention failed because the lesion could not be crossed, and an intra-aortic balloon pump was inserted via the femoral artery. The patient was taken for emergent coronary artery bypass grafting, which was uneventful. He was subsequently weaned from circulatory support and extubated. A postoperative echocardiogram demonstrated a mildly reduced left ventricular ejection fraction and mid-to-distal anterior and anteroseptal hypokinesis.

**Figure 1:**
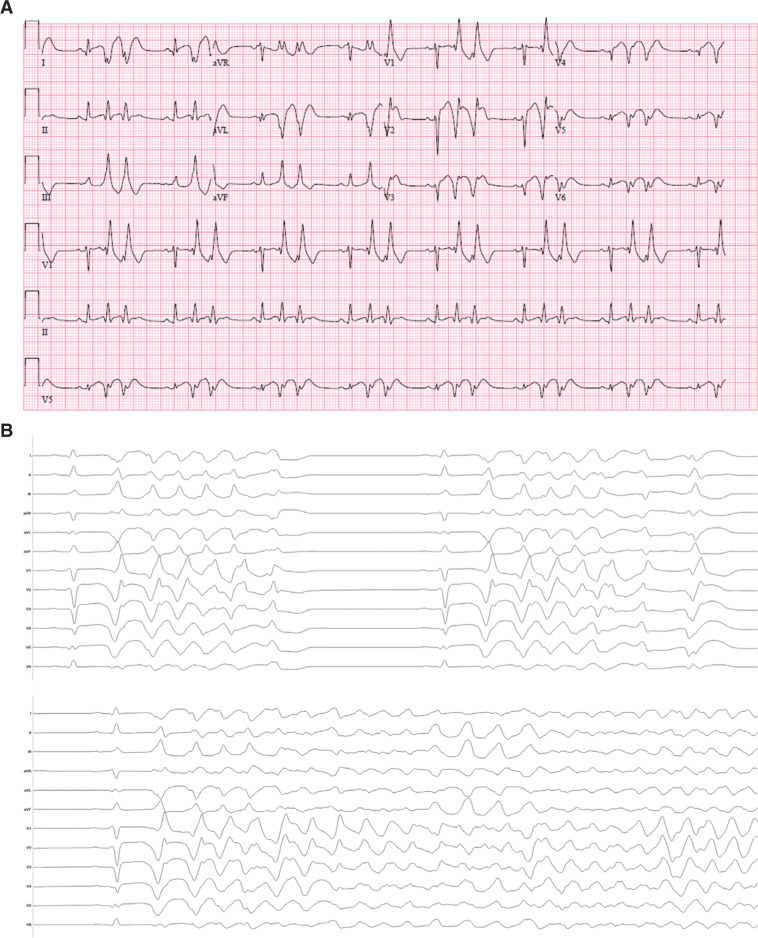
**A:** Preprocedural 12-lead ECG demonstrates frequent, short-coupled (< 300 ms), unifocal PVCs, suggestive of a His–Purkinje trigger. **B:** Frequent PVCs triggered both nonsustained ventricular tachycardia and VF.

On the third day after surgery, the patient began to experience symptomatic frequent PVCs **(Figure 1A)**. These were noted to trigger nonsustained polymorphic ventricular tachycardia (PMVT) that degenerated into VF, requiring defibrillation. Electrolytes were within their normal ranges and repeat coronary angiography revealed patent grafts and no occlusions. Frequent PVCs and VF continued despite intravenous boluses of metoprolol and continuous infusions of amiodarone, lidocaine, and procainamide. In light of the patient being in an immediate postinfarction state, we attempted additional temporizing measures in anticipation that his burden of arrhythmia might abate as more time elapsed. He was intubated and placed under general anesthesia with propofol and fentanyl but continued to experience recurrent VF. Ultrasound-guided percutaneous bedside bilateral percutaneous stellate ganglion blockade had no effect. He was cannulated for extracorporeal membranous oxygenation (ECMO) to provide circulatory support but continued to have intractable VAs.

**Figure 2:**
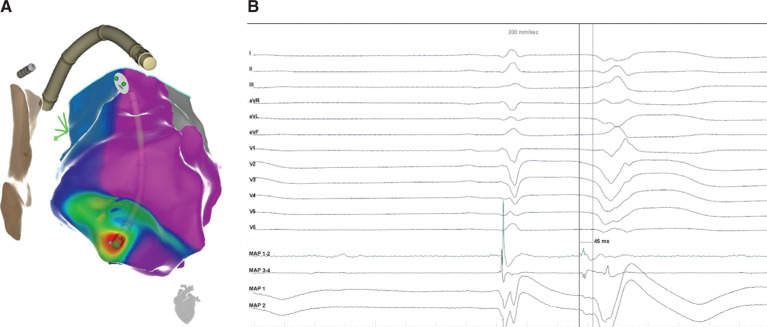
**A:** The site of origin of the triggering PVC was mapped to the anteroseptal left ventricular apex. **B:** Fractionated electrograms were 45 ms presystolic.

**Figure 3:**
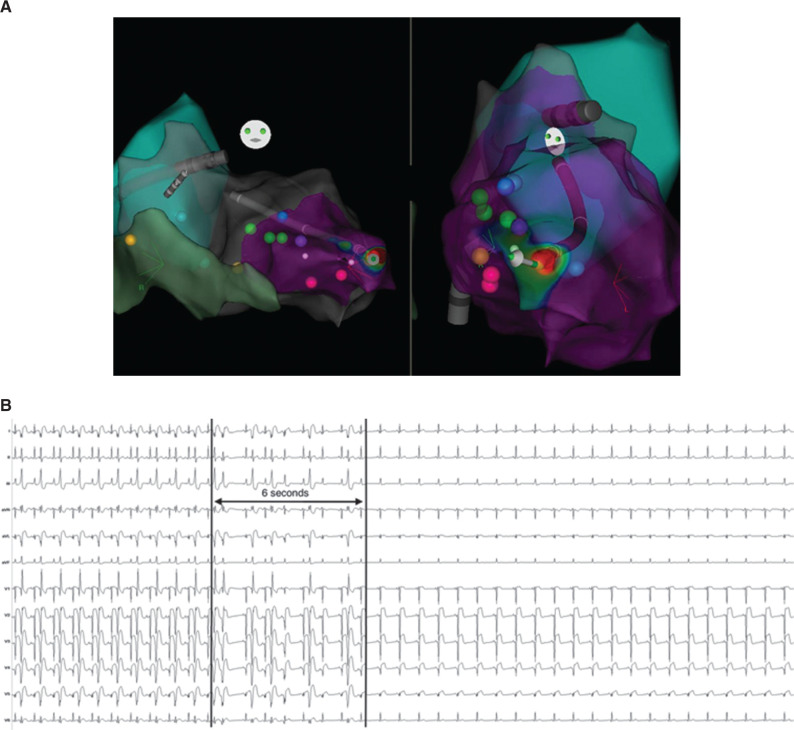
**A:** The PVC was localized to the anteroseptal left ventricular apex (anteropostetior projection on left, left anterior oblique projection on right). **B:** After six seconds of radiofrequency energy delivery at the indicated site, there were no further PVCs and no ventricular tachycardia or VF.

In light of his treatment-refractory VF storm and the apparent unifocal PVC trigger, we elected to bring him to the electrophysiology laboratory to undergo catheter ablation. We accessed the left ventricle with both transseptal and retrograde approaches. His presenting rhythm was sinus, with frequent PVCs that triggered PMVT, which would degenerate subsequently into ventricular flutter or VF **(Figure 1B)**, requiring frequent defibrillation. After each shock, we mapped the PVC trigger until his next VF episode. We eventually localized the site of origin to the anteroseptal left ventricular apex, where we found fractionated electrograms that were 45 ms pre-QRS **(Figures 2A and 2B)**. We delivered radiofrequency energy here and, after six seconds, the PVC was rendered quiescent **(Figures 3A and 3B)**. Postablation electrophysiologic study demonstrated no inducible arrhythmia, despite aggressive ventricular programmed stimulation. The remainder of his hospital stay was uneventful, he awoke with full neurological function, and he underwent placement of a secondary-prevention implantable cardioverter-defibrillator prior to being discharged home, with no arrhythmia occurring during follow-up despite not being on antiarrhythmic drug therapy.

## Discussion

Our patient experienced refractory VF storm (in the setting of acute ST-elevation myocardial infarction with failed percutaneous coronary intervention leading to emergent coronary artery bypass surgery) that persisted despite normal electrolyte levels, exclusion of ongoing ischemia, infusions of antiarrhythmic drugs, general anesthesia, full circulatory support with ECMO, and cardiac sympathetic denervation. Given that the VF appeared to always be triggered by a unifocal PVC, we performed catheter ablation, after which point the patient had no PVCs and no VA.

In reviewing this case, there are several key teaching points. First, not all VF is created equal. While VF represents a chaotic, disorganized ventricular electrical activity, the mechanism of progression from sinus rhythm to VF differs from patient to patient. Just as some atrial fibrillation is chiefly a problem of frequent ectopic triggers in an otherwise normal atrium but, in some patients, is characterized by end-stage atrial cardiopathy,^7^ VAs can behave similarly.

A manifested arrhythmia requires both a trigger and a vulnerable substrate. In a patient with a diffuse cardiomyopathic process such as nonischemic cardiomyopathy, the degree of dispersion of refractoriness and the number of circuits available for reentry are such that even a small burden of ectopic beats, serving as triggers, will induce arrhythmia, which is then easily sustained because of the diseased substrate. In contrast, our patient’s frequent VA was chiefly a problem of incessant triggers. As such, he is an ideal patient for a catheter ablative approach, and the recently published Heart Rhythm Society guidelines provide a class Ib recommendation for catheter ablation of postinfarction Purkinje fiber–mediated ventricular tachycardia.^8^

Second, the mechanism of arrhythmia initiation and maintenance can have profound implications on suitability for various treatments, so recognition is critical. Prior literature exploring catheter ablation for VF has centered around the His–Purkinje network.^5,6^ While the Purkinje fibers are known to be a trigger for idiopathic VF, emerging evidence suggests that the His–Purkinje network can serve as a trigger for VAs in patients with structural heart disease as well.^9^ In the postrevascularization period, anaerobic conditions and the inflammatory cascade characteristic of ischemia–reperfusion injury have potent effects on the cells of the His–Purkinje network, leading to membrane instability, deranged calcium handling, and enhanced automaticity.^10^ In our patient, the ECG characteristics and electroanatomic map were consistent with a trigger from the distal His–Purkinje network, just after arborization of the left anterior fascicle. Recognizing this ECG signature of short-coupled (< 300 ms) PVCs being suggestive of Purkinje ectopy, the burden on telemetric monitoring, and the reproducible relationship between the triggering PVC and the subsequent VF can provide guidance in patient selection for catheter ablation.

Third, to support successful catheter ablation of VF, the significance of full circulatory support with ECMO cannot be overstated. The acidemia and electrolyte derangements that would result from systemic malperfusion in the absence of circulatory support would alter the electrophysiological properties of the myocardium, which could hinder electroanatomic localization by potentiating additional arrhythmias. In addition, our patient had a cumulative burden of nonperfusing VA of several hours, requiring dozens of shocks, and the neurological sequelae of this duration of VA would be devastating in the absence of circulatory support. Alternative circulatory assist devices, such as intra-aortic balloon pump counterpulsation and nonballoon percutaneous mechanical circulatory support devices (eg, Impella; Abiomed, Danvers, MA, USA), might be considered as they are less resource-intensive than ECMO, but, given that the clinical arrhythmia of our patient was VF with no pulse pressure, partial assist devices such as these would likely provide inadequate support and may limit options for intraoperative mapping and ablation due to intolerance to the arrhythmia.

## Conclusions

This case serves as a reminder of three key teaching points. First, not all VF is created equal, with some instances being chiefly a problem of vulnerable substrate and others being best accounted for by frequent triggers. Second, examining the available ECG data and appropriately interpreting them can guide the selection of therapies up to and including catheter ablation for treatment-refractory VF. Third, full circulatory support greatly facilitates successful electroanatomic mapping and catheter ablation of unstable VAs.
